# The Evolving Role of Genetic Evaluation in the Prenatal Diagnosis and Management of Congenital Heart Disease

**DOI:** 10.3390/jcdd11060170

**Published:** 2024-05-30

**Authors:** Emily M. Bucholz, Sarah U. Morton, Erin Madriago, Amy E. Roberts, Christina Ronai

**Affiliations:** 1 Section of Cardiology, Department of Pediatrics, University of Colorado Denver, Denver, CO 80204, USA; 2 Division of Newborn Medicine, Department of Pediatrics, Boston Children’s Hospital, Harvard Medical School, Boston, MA 02115, USA; 3 Department of Pediatrics, Oregon Health and Science University, Portland, OR 97239, USA; 4 Department of Cardiology, Boston Children’s Hospital, Department of Pediatrics, Harvard Medical School, Boston, MA 02115, USA

**Keywords:** genetic testing, prenatal diagnosis, perinatal counseling, congenital heart disease

## Abstract

Congenital heart disease (CHD) is increasingly diagnosed prenatally and the ability to screen and diagnose the genetic factors involved in CHD have greatly improved. The presence of a genetic abnormality in the setting of prenatally diagnosed CHD impacts prenatal counseling and ensures that families and providers have as much information as possible surrounding perinatal management and what to expect in the future. This review will discuss the genetic evaluation that can occur prior to birth, what different genetic testing methods are available, and what to think about in the setting of various CHD diagnoses.

## 1. Introduction

Congenital heart disease (CHD) is the most common congenital defect [1] and is an important cause of morbidity and mortality, as it has an incidence of ~1% with a higher proportion in miscarriages [2]. The etiology of CHD is multifactorial and both environmental and genetic factors have been implicated [3,4]. Recent advancements in next-generation sequencing including exome sequencing have elucidated new genetic etiologies for CHD. In addition, there has been an increase in the use of prenatal genetic testing. Non-invasive testing screens for and invasive testing diagnoses genetic syndromes associated with CHD. When a genetic cause of CHD is identified prenatally, it can allow families to have a more complete picture of prognosis and it can also guide clinical management by potentially identifying the involvement of other organ systems. 

Prenatal invasive genetic testing can be performed as early as 10–11 weeks gestation via chorionic villus sampling (CVS) or later in pregnancy by amniocentesis. Increasingly, non-invasive prenatal testing (NIPT) is offered to obtain fetal cell-free DNA from a maternal blood sample to screen for aneuploidies (e.g., trisomy 21, 18, and 13, monosomy X) as well as common microdeletions and duplications (e.g., 22q11.2 microdeletion, Prader–Willi/Angelman). NIPT screening is often confirmed with CVS, amniocentesis, or postnatal genetic testing [5]. 

Understanding genetic contributions to CHD can help with risk stratification, prognosis (both short- and long-term), counseling, and postnatal management strategies. Multiple studies have demonstrated worse neurodevelopmental outcomes in CHD patients that also have a known genetic syndrome and/or extra cardiac anomalies [6,7,8,9]. In addition, prenatal genetic testing can be helpful in guiding both perinatal management and ongoing screenings for respiratory issues in patients found to have ciliary dyskinesia [10] or in those found to have genes that contribute to myocardial dysfunction and heart failure [11,12]. 

## 2. Common Genetic Syndromes in CHD

Currently, genetic causes can be identified in approximately 35% of CHD cases. Genetic causes of CHD can be grouped into aneuploidies, copy number variants, and single-gene variants. Aneuploidies were the earliest identified genetic causes of CHD and account for approximately 8–19% of CHD cases [13]. Approximately 35–50% of infants with trisomy 21 and 50–80% of infants with trisomy 13 and 18 have some form of CHD spanning a broad range of lesions.

Copy number variants (CNVs) refer to deletions or duplications of segments of chromosomes that can be inherited or occur de novo and can be detected through array-based platforms and exome or genome sequencing. CNVs underlie multiple cardiovascular genetic syndromes, such as 22q11.2 microdeletion (DiGeorge syndrome or velocardio-facial syndrome), deletion of 7q11.23 (Williams syndrome [14]), and 11q24-25 deletion (Jacobsen syndrome) [15,16], and are estimated to contribute to 10–15% of CHD [16].

Single-gene variants refer to changes in the nucleotide sequence of a particular gene that cause alterations in protein structure or function. These variants most commonly affect genes that encode cardiac transcription factors (e.g., NKX2.5, the GATA family of zinc finger proteins, T-box factors such as TBX5 or TBX1, ZIC3, and MEF2 factors), signaling molecules (e.g., NOTCH1, JAG1), or cellular structures (e.g., matrix metalloproteinases, cytoskeletal proteins). Current estimates suggest that approximately 3–5% of CHD is caused by inherited or de novo single-gene variants [9,17].

Extracardiac anomalies are common in genetic syndromes associated with CHD and often prompt referral to fetal cardiology when identified prenatally even before the diagnosis of CHD is made. The incidence of CHD when ≥1 extracardiac malformation is identified on prenatal screening is estimated to be between 20 and 45%, depending on the type of malformation [18,19]. Additionally, abnormalities in nuchal translucency and the umbilical cord vasculature have been associated with an increased risk of genetic abnormalities and CHD and thus are indications for referral to fetal cardiology. While it is not possible to summarize all known genetic associations with CHD, we have highlighted common aneuploidies, CNVs, and single-gene variants associated with CHD and common non-cardiac manifestations in Table 1.

## 3. Genetic Counseling

For families, a prenatal diagnosis of CHD frequently leads to many questions not only about the current pregnancy but also about the future. Concerns around the potential cause of the disease and, if genetic, the likelihood of recurrence with subsequent pregnancies are frequent. Questions about what a genetic diagnosis means for the pregnant patient or partner and whether other family members should be screened can be complex. At present, systematic genetic screening of first-degree relatives is not recommended for non-syndromic CHD outside of patients with left-sided obstructive lesions and bicuspid aortic valve. However, when genetic syndromes such as Noonan syndrome or 22q11.2 microdeletion are identified, the screening of parents and/or siblings should be discussed. Additionally, patients who are contemplating future pregnancies should also be counseled on the availability and potential utility of carrier screening when a genetic anomaly is identified and the residual risk of having a second affected pregnancy even when genetic testing has been non-diagnostic.

Most frequently, a genetic diagnosis leads to questions about the fetal prognosis, both short and long term [20,21,22,23]. Nuanced conversations about the overarching potential impact of a genetic diagnosis on management, quality of life, and neurodevelopment along with phenotypic variability of the diagnosis can be challenging but is essential. Immediate access to prenatal genetic counseling with initial diagnosis enables these conversations to take place early on, allowing time for information to be absorbed and for families to prepare for postnatal life. This information can be valuable to individuals in determining significant life choices, including but not limited to continuation of the pregnancy or the role for comfort care after birth. Additionally, it provides an opportunity to talk about other potential needs that may arise during postnatal care. Families may benefit from the time to absorb and process this information prior to delivery and outside of the intensive care unit. Knowledge about medical needs empowers families to partner with the team and participate in decision making at a time when they may feel as if they have no choices. A coordinated team approach including maternal fetal medicine, cardiology, and genetics is likely to be most helpful to the family.

## 4. Prenatal Genetic Testing

Prenatal genetic testing falls into two broad categories: screening or diagnostic. Screening genetic testing includes non-invasive prenatal testing (NIPT) of cell-free fetal DNA (cfDNA) present in the maternal circulation, which is typically possible starting at 9–10 weeks of gestation [24,25]. The genetic variants detected by NIPT depend on the type of sequencing and data analysis that is performed. Most versions of NIPT offered commercially screen for trisomy 13, 18, and 21 as well as sex chromosome aneuploidies [26,27,28]. Some versions also screen for specific deletions and duplications such as 22q11.2 microdeletion, large deletions and duplications genome-wide, or specific single-gene disorders [29,30,31].

Diagnostic prenatal genetic testing can be performed on cells of fetal origin, including placental tissue from chorionic villus sampling (CVS) or amniotic fetal cells from amniocentesis [32]. Fetal DNA can then be characterized by genetic testing including fluorescence in situ hybridization (FISH), chromosomal microarray (CMA), gene panel, exome sequencing, or genome sequencing. FISH uses targeted probes that are visualized using microscopy of metaphase chromosomes to detect missing or duplicated DNA at a specific region of interest such as for 22q11.2 deletion [33]. Chromosomal microarray involves the hybridization of patient DNA sequences to a set of known sequences that allows for the detection of genomic regions that have deletions or duplications [34,35]. Gene panels and exome sequencing typically involve the determination of the specific nucleotides using the direct sequencing of patient DNA [36,37,38,39,40]. Genome sequencing has the benefit of identifying structural variation that can be detected by CMA as well as changes at the nucleotide level of coding and non-coding DNA across the genome. The resultant data are then aligned to the reference human genome to determine changes in DNA sequences that indicate single nucleotide changes, small insertions/deletions, or larger duplications/deletions. Parental sequencing can be particularly useful to interpret the results of exome or genome sequencing, as many individuals have rare inherited variants that would be interpreted as less likely to cause disease if present in an unaffected parent.

## 5. Postnatal Genetic Testing in CHD

Our ability to find a specific molecular genetic etiology for CHD has improved over time as new genes have been identified and new genome-wide analysis technologies have become clinically available. The AHA (endorsed by the AAP) published updated guidelines in 2018 describing the need for genetic testing in CHD due to increasing clinical relevance to uncover associated extra-cardiac issues, understand the risk of neurodevelopmental delays, refine recurrence risk estimates for parents and the individual with CHD, and provide prognostic information [17]. For infants requiring early surgical intervention, studies have shown that positive genetic testing results have been associated with post-operative complications and survival and can impact care and resource utilization [16,41,42,43,44,45]. Cohort studies have demonstrated that genetic testing of newborns with CHD yields an overall diagnostic rate of ~30% with genome-wide analysis [46,47,48,49,50]. The testing yield is slightly higher in cases of CHD with extra-cardiac anomalies, but results are still robust when the CHD is isolated [51,52]. In 2021, the American College of Medical Genetics published an evidence-based clinical guideline recommending exome or genome sequencing as a first- or second-tier test for children with congenital anomalies, including CHD [53]. Exome and genome data can be re-analyzed at future time points to look for variants in CHD genes described since the time of the first analysis.

The decision to perform genetic testing postnatally depends on multiple factors. Diagnostic genetic testing should be offered if NIPT screened in for a genetic diagnosis and no follow-up testing was completed prior to birth, non-cardiac anomalies were found prenatally or identified postnatally, if there is a family history of CHD, or if a genetic diagnosis is strongly suspected based on the particular heart disease, facial features, or pattern of heart disease and non-cardiac anomalies.

When a genetic syndrome is suspected based on a neonate’s outward physical appearance or the presence of classic CHD lesions (e.g., conotruncal abnormalities for 22q11.2 microdeletion or supravalvar aortic or pulmonary stenosis for Williams syndrome), a comprehensive clinical examination is essential for identifying extracardiac involvement. This includes the assessment of common physical findings (e.g., facial dysmorphia, limb length discrepancies, hand and feet anomalies, skeletal abnormalities), growth delays, and additional imaging as needed to exclude gastrointestinal, urologic, and genital defects. This approach to screening often involves a multidisciplinary team but can be essential for identifying additional defects that require ongoing management and can help guide genetic testing when a probable syndrome phenotype is identified.

A strong family history can also prompt genetic testing when there are first- or second-degree relatives with diagnosed CHD. Comprehensive questioning of past medical and surgical histories of family members should be used to generate a family pedigree to identify possible inheritance patterns. It is important to note that different phenotypes related to the same genetic mutation may be expressed across generations. Such pedigrees can be used to guide genetic testing and counsel family members on the likelihood of recurrence under the guidance of a genetic specialist.

Finally, parental preference is essential to consider when deciding whether and how genetic testing will be performed. For some families, the presence of a genetic diagnosis will not change management or decision making and thus they prefer not to seek additional genetic work up. Such preferences must be respected but should be frequently re-evaluated particularly if the clinical picture changes, the parents are considering another pregnancy, and/or when children reach adulthood and are capable of making decisions around their own care and family planning.

Taken together, this evidence strongly supports offering exome- or genome-wide sequencing for neonates with isolated or syndromic CHD. One may consider focused genetic testing in phenotypes highly suggestive of a particular diagnosis (e.g., chromosomal microarray to evaluate for 22q11.2 deletion in tetralogy of Fallot with pulmonary atresia or interrupted aortic arch type B or 7q11.23 FISH to evaluate for Williams syndrome in supravalvar aortic stenosis). If genome or exome sequencing is not available, structural heart disease gene panel testing and chromosomal microarray is a strong second option. The benefits of testing increase when offered earlier in the diagnostic evaluation to shorten the time to a diagnosis and targeted therapeutic interventions [53,54].

There is currently a list of 81 genes that the American College of Medical Genetics recommends offering analysis of when exome or genome sequencing is completed [55]. The 81 genes are each associated with conditions for which there are clear management or surveillance guidelines. The conditions analyzed include cancer predisposition syndromes, inherited arrhythmias, cardiomyopathies, inborn errors of metabolism, and several others. Some of the conditions are adult onset while others can present in childhood. Because these secondary findings are unrelated to why testing may have been pursued in the first place, this type of result is optional to receive. Genetic counseling prior to initiating this testing is required to obtain informed consent from those tested as to whether or not they would like to have these additional results.

## 6. Conclusions

In summary, prenatal genetic testing can be invaluable to families with prenatally diagnosed CHD in order to assist with decision making surrounding delivery, involvement of other organ systems, and planning for the future. When families decline invasive prenatal testing, postnatal genetic testing can ensure that families and providers have as much information as possible to support neonates both through possible cardiac interventions and throughout their childhood.

## Figures and Tables

**Table 1:** Common aneuploidies, copy number variants, and single-gene variants associated with CHD and common non-cardiac manifestations.

Mutation	Incidence of CHD	Associated CHD	Non-Cardiac Malformations and Features	Detectable by Testing Method				
				K	CMA	GPS	ES	GS
**Aneuploidies**								
Trisomy 13 (Patau)	60–80%	ASD, VSD, PDA, TOF, PHTN	Facial dysmorphism, cleft lip/palate, CNS abnormalities, polydactyly, GI/GU anomalies	x	x		x	x
Trisomy 18 (Edward)	60–80%	ASD, VSD, PDA, PHTN	Facial dysmorphism, hypotonia, growth retardation, rocker-bottom feet, clenched hands, GI/GU anomalies, CNS abnormalities, IUGR	x	x		x	x
Trisomy 21 (Down)	35–50%	AVSD, VSD, ASD, PDA	Facial dysmorphism, GI anomalies, hypothyroidism, hypotonia, vertebral anomalies	x	x		x	x
Monosomy X (Turner)	25–45%	Left-sided obstructive lesions (CoA, BAV, AS)	Webbed neck, shield chest, renal anomalies, lymphedema, ovarian dysgenesis, cystic hygroma (often with internal septation), nonimmune hydrops	x	x		x	x
4p- (Wolf Hirschhorn)	30–80%	ASD	Facial dysmorphism, scoliosis, cleft lip/palate, seizures, intellectual disability	x	x		x	x
5p- (Cri de Chat)	5–30%	VSD, TOF, PA, DORV	Microcephaly, hypotonia, high-pitched cry	x	x		x	x
**Copy Number Variants**								
22q11.2 deletion (DiGeorge)	75%	Conotruncal anomalies (TOF, IAA, truncus arteriosus)	Cleft palate, developmental delays, facial dysmorphism, renal anomaly, absent thymus		x		x	x
7q11.23 deletion (Williams)	75–80%	Supravalvar AS, branch PS, VSD	Arterial stenosis, facial dysmorphism, intellectual disability, IUGR		x		x	x
11q24-25 deletion (Jacobsen)	55%	Left-sided obstructive lesions, VSD	Facial dysmorphism, skull abnormality, bleeding disorder, GI/GU abnormalities		x		x	x
**Single-Gene Changes**								
Rasopathies (Noonan, cardiofaciocutaneous, and Costello syndromes) (*BRAF*, *HRAS*, *KRAS*, *LZTR1*, *MAP2K1*, *MAP2K2*, *MRAS*, *NRAS*, *PTPN11*, *RAF1*, *RASA2*, *RIT1*, *RRAS2*, *SHOC2*, *SOS1*, *SOS2*)	70–80%	PS, ASD, hypertrophic cardiomyopathy, VSD, AVSD, CoA	Facial dysmorphism, short stature, neurodevelopmental delay, increased nuchal translucency, cystic hygroma, polyhydramnios, absent ductus venosus			x	x	x
Alagille syndrome *JAG1*, *NOTCH2*	90%	PPS, PS, TOF	Cholestasis, skeletal abnormalities, eye, facial dysmorphism			x	x	x
Kabuki syndrome *KMT2D*, *KDM6A*	30–55%	Left-sided obstructive lesions, HLHS, CoA	Polyhydramnios, GU anomalies, single umbilical artery, IUGR, hydrops/pleural effusion/ascites			x	x	x
CHARGE syndrome *CHD7*	75–85%	TOF, DORV, ASD, VSD	Dandy-Walker malformation, holoprosencephaly, choanal atresia, cleft lip/palate, micrognathia, esophageal atresia/stenosis, omphalocele, renal anomalies			x	x	x
Ellis van Creveld syndrome *EVC/EVC2*	50–60%	Common atrium, atrioventricular valve dysplasia, PDA, HLHS	Cleft lip/palate, cryptorchidism, short long bones, narrow thorax, hand anomalies, peg teeth			x	x	x
Smith–Lemi–Opitz syndrome *DHCR7*	50%	AVSD, PAPVR	Facial dysmorphism, short stature, cleft palate, hypotonia, intellectual disability, hand anomalies, renal anomalies			x	x	x
Heterotaxy *ACVR2B*, *ARMC4*, *CCDC103*, *CCDC114*, *CCDC151*, *CCDC39*, *CCDC40*, *CCDC65*, *CCNO*, *CFAP298*, *CFAP300*, *CFAP53*, *CRELD1*, *CFC1*, *DNAAF1*, *DNAAF2*, *DNAAF3*, *DNAAF4*, *DNAAF5*, *DNAH1*, *DNAH11*, *DNAH5*, *DNAH9*, *DNAI1*, *DNAI2*, *DNAJB13*, *DNAL1*, *DRC1*, *FOXH1*, *FOXJ1*, *GDF1*, *GAS2L2*, *GAS8*, *HYDIN*, *LEFTY1*, *LEFTY2*, *LRRC56*, *LRRC6*, *MCIDAS*, *MMP21*, *MNS1*, *NEK10*, *NME8*, *NKX2.5*, *NODAL*, *PIH1D3*, *PKD1L1*, *RSPH1*, *RSPH3*, *RSPH4A*, *RSPH9*, *SPAG1*, *STK36*, *TP73*, *TTC12*, *TTC25*, *ZIC3*, *ZMYND10*	80%	TAPVR, PAPVR, atrial situs ambiguous or inversus, CAVC, HLHS, DORV, TGA, PAIVS, dextrocardia, bilateral SVC, interrupted IVC	Biliary atresia, abdominal situs abnormalities, asplenia/polysplenia, lung isomerism, intestinal malrotation, absent gallbladder, primary ciliary dyskinesia			x	x	x
Underline refers to anomalies that can be detected on screening prenatal ultrasound. K: karyotype, CMA: chromosomal microarray, GPS: gene panel sequencing, ES: exome sequencing, GS: genome sequencing, IUGR: intrauterine growth restriction, CNS: central nervous system, GI: gastrointestinal, GU: genitourinary. ASD: atrial septal defect, VSD: ventricular septal defect, PDA: patent ductus arteriosus, TOF: tetralogy of Fallot, PHTN: pulmonary hypertension, AVSD: atrioventricular canal defect, CoA: coarctation of the aorta, BAV: bicuspid aortic valve, AS: aortic stenosis, PA: pulmonary atresia, DORV: double-outlet right ventricle, IAA: interrupted aortic arch, PS: pulmonary stenosis, HLHS: hypoplastic left heart syndrome, PAPVR: partial anomalous pulmonary venous return, TAPVR: total anomalous pulmonary venous return, TGA: transposition of the great arteries, PAIVS: pulmonary atresia intact ventricular septum, SVC: superior vena cava, IVC: inferior vena cava.								

## Data Availability

Data sharing is not applicable.
